# Hesitant Triangular Fuzzy Information Aggregation Operators Based on Bonferroni Means and Their Application to Multiple Attribute Decision Making

**DOI:** 10.1155/2014/648516

**Published:** 2014-07-16

**Authors:** Chunyong Wang, Qingguo Li, Xiaoqiang Zhou, Tian Yang

**Affiliations:** ^1^College of Mathematics and Econometrics, Hunan University Changsha, Hunan 410082, China; ^2^College of Mathematics, Hunan Institute of Science and Technology, Yueyang, Hunan 414006, China; ^3^College of Science, Central South University of Forestry and Technology, Changsha, Hunan 410004, China

## Abstract

We investigate the multiple attribute decision-making (MADM) problems with hesitant triangular fuzzy information. Firstly, definition and some operational laws of hesitant triangular fuzzy elements are introduced. Then, we develop some hesitant triangular fuzzy aggregation operators based on Bonferroni means and discuss their basic properties. Some existing operators can be viewed as their special cases. Next, we apply the proposed operators to deal with multiple attribute decision-making problems under hesitant triangular fuzzy environment. Finally, an illustrative example is given to show the developed method and demonstrate its practicality and effectiveness.

## 1. Introduction

Fuzzy set (FS), proposed by Zadeh in 1965 [1], has achieved a great success in various fields since it appears. As extensions of FS, the intuitionistic fuzzy set and interval-valued intuitionistic fuzzy set have received much attention [2–6]. Furthermore, Torra [7] generalized FS to hesitant fuzzy set (HFS), which allows the membership to a set represented by several possible values. HFS is very useful to express people's hesitancy in daily life and a series of aggregation operators for hesitant fuzzy information have been developed [8–13]. Although HFS is a powerful tool to deal with uncertainty, it still has inherent drawbacks. HFS only permits the membership having a set of possible exact and crisp values. However, due to the increasing complexity of the socioeconomic environment and the vagueness of inherent subjective nature of human think, the information provided by a decision-maker is often imprecise or uncertain, so exact and crisp values are usually insufficient to model real-life decision. Chen et al. [14] introduced the interval-valued hesitant fuzzy set, based on which Wei et al. [15] proposed the hesitant triangular fuzzy set. As we all know, triangular fuzzy number is a very suitable tool to express uncertainty. Hesitant triangular fuzzy set (HTFS), whose membership degrees are expressed by several possible triangular fuzzy numbers, is more adequate or sufficient to solve real-life decision problem than real numbers. For example [16], suppose three reviewers are to estimate the degrees that a candidate satisfies the criterion of honest. As they have not met each other before, the evaluation is uncertain. The first reviewer thinks the most possible of the candidate satisfying the criterion of honest is 0.8, the minimum possible is 0.7, and the maximum possible is 0.9. Then, he can give the evaluation by a triangular fuzzy number (0.7,0.8,0.9). Similarly, the second reviewer and the third reviewer give their evaluations as (0.4,0.5,0.6) and (0.6,0.9,1.0), respectively. As a result, this comprehensive evaluation can be expressed by a hesitant triangular fuzzy element {(0.7,0.8,0.9), (0.4,0.5,0.6), (0.6,0.9,1.0)}. In this case, HTFS describes the dilemma vividly.

In [15], Wei et al. also developed some hesitant triangular fuzzy aggregation operators such as the hesitant triangular fuzzy weighted averaging (HTFWA) operator, hesitant triangular fuzzy weighted geometric (HTFWG) operator, hesitant triangular fuzzy ordered weighted averaging (HTFOWA) operator, hesitant triangular fuzzy ordered weighted geometric (HTFOWG) operator, hesitant triangular fuzzy hybrid averaging (HTFHA) operator, and hesitant triangular fuzzy hybrid geometric (HTFHG) operator.

However, the above operators, which are the extensions of the average mean (AM) and the geometric mean (GM), only consider the situations where all the elements are independent. Luckily, the Bonferroni mean (BM), which is originally introduced by Bonferroni [17] and generalized by Yager [18], can capture the interrelationships among arguments [17, 19–24]. Moreover, the Choquet integral is also an important tool to consider the correlations among attributes [6, 25–27]. With the analysis above, we attempt to develop new hesitant triangular fuzzy aggregation operators based on the BM and the Choquet integral so as to capture both the interrelationships between input arguments and the correlations among the attributes.

To facilitate our discussion, the remainder of this paper is organized as follows. In the next section, we review some basic concepts. Hesitant triangular fuzzy geometric Bonferroni mean operator and its properties are studied in Section 3. In Section 4, families of hesitant triangular fuzzy aggregation operators based on BM are studied. The relations between these new operators and the existing operators are also investigated. In Section 5, we develop a method for multiple attribute decision-making based on new operators under hesitant triangular fuzzy environment. An illustrative example is also given to show the effectiveness of the developed approach in Section 6. In Section 7, we conclude the paper and give some remarks.

## 2. Preliminaries

### 2.1. Triangular Fuzzy Numbers and Hesitant Triangular Fuzzy Set

FS was first proposed by Zadeh [1] in 1965.

**Definition 1 (see [1]).**

Let *X* be an universe of discourse; then a fuzzy set is defined as *A* = {〈*x*, *μ*_*A*_(*x*)〉∣*x* ∈ *X*} which is characterized by a membership function *μ*_*A*_ : *X* → [0,1], where *μ*_*A*_ denotes the degree of membership of the element *x* to the set *A*.

Torra [7] generalized FSs to HFSs as follows.

**Definition 2 (see [7]).**

Let *X* be a reference set; then one defines hesitant fuzzy set on *X* in terms of a function that when applied to *X* returns a sunset of [0,1].

To be easily understood, Xia and Xu [8] express the HFS by a mathematical symbol: *E* = (〈*x*, *h*_*E*_(*x*)〉∣*x* ∈ *X*), where *h*_*E*_(*x*) is a set of some values in [0,1], denoting the possible membership degree of the element *x* ∈ *X* to the set *E*. For convenience, Xia and Xu [8] call *h* = *h*_*E*_(*x*) a hesitant fuzzy element (HFE) and *H* the set of all HFEs when there is no confusion.

Triangular fuzzy number, which is proposed by van Laarhoven and Pedrycz [28], is also a useful tool to express uncertainty.

**Definition 3 (see [28]).**

A triangular fuzzy number $\tilde{a}$ can be defined by a triplet (*a* ^*L*^, *a* ^*M*^, *a* ^*U*^). The membership function $\mu_{\tilde{a}}(x)$ is defined as

(1) $$\begin{array}{c} \mu_{\tilde{a}}\left(x\right)=\{\begin{array}{cc} 0, & x<a^{L},\\ \frac{x-a^{L}}{a^{M}-a^{L}}, & a^{L}\leq x\leq a^{M},\\ \frac{x-a^{U}}{a^{M}-a^{U}}, & a^{M}\leq x\leq a^{U},\\ 0, & x\geq a^{U}, \end{array} \end{array}$$

where 0 < *a* ^*L*^ ≤ *a* ^*M*^ ≤ *a* ^*U*^, *a* ^*L*^ and *a* ^*U*^ stand for the lower and upper values of the support of $\tilde{a}$, respectively, and *a* ^*M*^ stands for the modal value.

In [28], basic operational laws related to triangular fuzzy numbers were also given as

 
$\tilde{a}⊕\tilde{b}=[a^{L},a^{M},a^{U}]⊕[b^{L},b^{M},b^{U}]=[a^{L}+b^{L},a^{M}+b^{M},a^{U}+b^{U}]$;
 
$\tilde{a}⊗\tilde{b}=[a^{L},a^{M},a^{U}]⊗[b^{L},b^{M},b^{U}]=[a^{L}b^{L},a^{M}b^{M},a^{U}b^{U}]$;
 
$\lambda ⊗\tilde{a}=\lambda ⊗[a^{L},a^{M},a^{U}]=[\lambda a^{L},\lambda a^{M},\lambda a^{U}]$,  *λ* > 0.

In order to compare triangular fuzzy numbers, many ranking methods have been proposed and each method has its advantages as well as drawbacks [29]. We adopt one of them as below.

**Definition 4 (see [30]).**

Let $\tilde{b}=[b^{L},b^{M},b^{U}]$ and $\tilde{a}=[a^{L},a^{M},a^{U}]$ be two triangular fuzzy numbers; then the degree of possibility of $\tilde{a}\geq \tilde{b}$ is defined as

(2) p(a~≥b~)  =λ max⁡{1−max⁡⁡[(bM−aL)(aM−aL+bM−bL),0],0}   +(1−λ)    ×max⁡⁡{1−max⁡⁡[(bU−aM)(aU−aM+bU−bM),0],0},

where the value *λ* is an index of rating attitude. It reflects the decision-maker's risk-bearing attitude. If *λ* > 0.5, the decision-maker is risk lover. If *λ* = 0.5, the decision-maker is neutral to risk. If *λ* < 0.5, the decision-maker is risk averter.

From this definition, we can get the following results easily:

1. $0\leq p(\tilde{a}\geq \tilde{b})\leq 1,0\leq p(\tilde{b}\geq \tilde{a})\leq 1$;
2. $p(\tilde{a}\geq \tilde{b})+p(\tilde{b}\geq \tilde{a})=1$. Especially, $p(\tilde{a}\geq \tilde{a})=p(\tilde{b}\geq \tilde{b})=0.5$.

Wei et al. [15] generalized the HFS to HTFS as follows.

**Definition 5 (see [15]).**

Let *X* be a fixed set; a hesitant triangular fuzzy set (HTFS) on *X* is in terms of a function when applied to each *x* in *X* and returns a subset of values in [0,1].

To be easily understood, Wei et al. [15] express the HTFS by a mathematical symbol: $E=\left\{\langle x,{\tilde{h}}_{E(x)}\rangle |x\in X\right\}$, where ${\tilde{h}}_{E(x)}$ is a set of some possible triangular fuzzy values in [0,1], denoting the possible membership degrees of the element *x* ∈ *X* to the set *E*. For convenience, they also call ${\tilde{h}}_{E(x)}$ a hesitant triangular fuzzy element (HTFE) and $\tilde{H}$ the set of all HTFEs.

Given three HTFEs $\tilde{h}$, $\tilde{h_{1}}$, $\tilde{h_{2}}$ and *λ* > 0, Wei et al. [15] defined their operations as follows:

1. ${\tilde{h}}^{\lambda }=\cup_{\gamma \in \tilde{h}}\{({\left(\gamma^{L}\right)}^{\lambda },{\left(\gamma^{M}\right)}^{\lambda },{\left(\gamma^{R}\right)}^{\lambda })\}$;
2. $\lambda \tilde{h}=\cup_{\gamma \in \tilde{h}}\{(1-{\left(1-\gamma^{L}\right)}^{\lambda },1-{\left(1-\gamma^{M}\right)}^{\lambda },1-{\left(1-\gamma^{R}\right)}^{\lambda })\}$;
3. ${\tilde{h}}_{1}⊕{\tilde{h}}_{2}=\cup_{\gamma_{1}\in {\tilde{h}}_{1},\gamma_{2}\in {\tilde{h}}_{2}}\{(\gamma_{1}^{L}+\gamma_{2}^{L}-\gamma_{1}^{L}\gamma_{2}^{L},\gamma_{1}^{M}+\gamma_{2}^{M}-\gamma_{1}^{M}\gamma_{2}^{M},\gamma_{1}^{R}+\gamma_{2}^{R}-\gamma_{1}^{R}\gamma_{2}^{R})\}$;
4. ${\tilde{h}}_{1}⊗{\tilde{h}}_{2}=\cup_{\gamma_{1}\in {\tilde{h}}_{1},\gamma_{2}\in {\tilde{h}}_{2}}\{(\gamma_{1}^{L}\gamma_{2}^{L},\gamma_{1}^{M}\gamma_{2}^{M},\gamma_{1}^{R}\gamma_{2}^{R})\}$.

In order to compare two HTFEs, the score function was defined as follows.

**Definition 6 (see [15]).**

For a HTFE $\tilde{h}$, $s(\tilde{h})=\left(1/♯h\right)\sum_{\tilde{\gamma }\in \tilde{h}}\tilde{\gamma }$ is called the score function of $\tilde{h}$, where $♯\tilde{h}$ is the number of the triangular fuzzy values in $\tilde{h}$ and $s(\tilde{h})$ is a triangular fuzzy value belonging to [0,1]. For two HTFEs ${\tilde{h}}_{1}$ and ${\tilde{h}}_{2}$, if $s({\tilde{h}}_{1})\geq s({\tilde{h}}_{2})$, then ${\tilde{h}}_{1}\geq {\tilde{h}}_{2}$.

### 2.2. Choquet Integral and Bonferroni Mean

In order to weight the elements in *X*, a fuzzy measure *μ* was defined as follows.

**Definition 7 (see [31]).**

A fuzzy measure *μ* on the set *X* is a set function *μ* : *θ*(*X*)→[0,1] satisfying the following axioms and *θ*(*X*) is the set of all subsets of *X*:

1. *μ*(*ϕ*) = 0, *μ*(*X*) = 1;
2. *A*⊆*B* implies *μ*(*A*) ≤ *μ*(*B*), for all *A*, *B*⊆*X*;
3. *μ*(*A* ∪ *B*) = *μ*(*A*) + *μ*(*B*) + *ρμ*(*A*)*μ*(*B*), for all *A*, *B*⊆*X* and *A*∩*B* = *ϕ*, where *ρ* ∈ (−1, *∞*).

Especially, if *ρ* = 0, then condition (3) reduces to the axiom of additive measure: *μ*(*A* ∪ *B*) = *μ*(*A*) + *μ*(*B*), for all *A*, *B*⊆*X* and *A*∩*B* = *ϕ*. If all the elements in *X* are independent, then we have *μ*(*A*) = ∑_*x*_*i*_∈*A*_ *μ*({*x*_*i*_}), ∀*A*⊆*X*.

The discrete Choquet integral is a linear expression up to a reordering of the elements.

**Definition 8 (see [32]).**

Let *f* be a positive real-valued function on *X*, and let *μ* be a fuzzy measure on *X*. The discrete Choquet integral of *f* with respect to *μ* is defined by

(3) $$\begin{array}{c} C_{\mu }\left(f\right)=\overset{n}{\underset{i=1}{\sum }}f_{\sigma (i)}\left[\mu \left(A_{\sigma (i)}\right)-\mu \left(A_{\sigma (i-1)}\right)\right], \end{array}$$

where (*σ*(1), *σ*(2),…, *σ*(*n*)) is a permutation of (1,2,…, *n*), such that *f*_*σ*(*i*−1)_ ≥ *f*_*σ*(*i*)_ for all *i* = 2,3,…, *n*, *A*_*σ*(*k*)_ = {*x*_*σ*(*j*)_∣*j* ≤ *k*}, for *k* ≥ 1, and *A*_*σ*(0)_ = *ϕ*.

As extensions of the arithmetic average and the geometric mean, the Bonferroni mean (BM) and the geometric Bonferroni mean (GBM) are very practical aggregation operators, which consider the interrelationships among arguments. We review the two operators as follows.

**Definition 9 (see [17, 33]).**

Let *p*, *q* ≥ 0 and let *a*_*i*_  (*i* = 1,2,…, *n*) be a collection of nonnegative numbers. Then

(4) $$\begin{array}{c} {\text{BM}}^{p,q}\left(a_{1},a_{2},\ldots ,a_{n}\right)={\left(\frac{1}{n(n-1)}\overset{n}{\underset{i,j=1,i\neq j}{\sum }}a_{i}^{p}a_{j}^{q}\right)}^{1/\left(p+q\right)}\\ {\text{GBM}}^{p,q}\left(a_{1},a_{2},\ldots ,a_{n}\right)=\frac{1}{p+q}\overset{n}{\underset{i\neq j,i,j=1}{\prod }}{\left(pa_{i}+qa_{j}\right)}^{1/n(n-1)}, \end{array}$$

are called Bonferroni mean (BM) [17] and geometric Bonferroni mean (GBM) [33], respectively.

### 2.3. The Existing Hesitant Triangular Fuzzy Operators

Here, we will briefly recall the existing hesitant triangular fuzzy operators. To see more details, we can refer to [15, 34].

Let *h*_*i*_  (*i* = 1,2,…, *n*) be a collection of HTFEs, let *p*, *q* ≥ 0, and let *w* = (*w*_1_, *w*_2_,…, *w*_*n*_)^*T*^ be the weight of *h*_*i*_, where *w*_*i*_ denotes the importance degree of *h*_*i*_, satisfying *w*_*i*_ > 0 and ∑_*i*=1_^*n*^ *w*_*i*_ = 1. Then the hesitant triangular fuzzy weighted averaging (HTFWA) operator was defined as [15] HTFWA(*h*_1_, *h*_2_,…, *h*_*n*_) = ⨁_*j*=1_^*n*^(*w*_*j*_ *h*_*j*_) and the hesitant triangular fuzzy weighted geometric (HTFWG) operator was defined as [15] HTFWG(*h*_1_, *h*_2_,…, *h*_*n*_) = ⨂_*j*=1_^*n*^(*h*_*j*_)^*w*_*j*_^. Furthermore, suppose (*σ*(1), *σ*(2),…, *σ*(*n*)) is a permutation of (1,2,…, *n*), such that *h*_*σ*(*j*−1)_ ≥ *h*_*σ*(*j*)_ for all *j* = 2,3,…, *n*. Then, the hesitant triangular fuzzy ordered weighted averaging (HTFOWA) operator was defined as [15] HTFOWA(*h*_1_, *h*_2_,…, *h*_*n*_) = ⨁_*j*=1_^*n*^(*w*_*j*_ *h*_*σ*(*j*)_) and the hesitant triangular fuzzy ordered weighted geometric (HTFOWG) operator was defined as [15] HTFOWG(*h*_1_, *h*_2_,…, *h*_*n*_) = ⨂_*j*=1_^*n*^(*h*_*σ*(*j*)_)^*w*_*j*_^.

Suppose ${\tilde{h}}_{\sigma (j)}$ is the *j*th largest element of the hesitant triangular fuzzy arguments (${\tilde{h}}_{j}=(nw_{j})h_{j},j=1,2,\ldots ,n$); then $\text{HTFHA}(h_{1},h_{2},\ldots ,h_{n})={⨁}_{j=1}^{n}(w_{j}{\tilde{h}}_{\sigma (j)})$ is called the hesitant triangular fuzzy hybrid average (HTFHA) operator [15].

Suppose ${\tilde{h}}_{\sigma (j)}$ is the *j*th largest element of the hesitant triangular fuzzy arguments (${\tilde{h}}_{j}={\left(h_{j}\right)}^{{nw}_{j}},j=1,2,\ldots ,n$); then $\text{HTFHG}(h_{1},h_{2},\ldots ,h_{n})={⨂}_{j=1}^{n}{\left({\tilde{h}}_{\sigma (j)}\right)}^{w_{j}}$ is called the hesitant triangular fuzzy hybrid geometric (HTFHG) operator [15].

Let *μ* be a fuzzy measure on *X*; HTFCOA_*μ*_(*h*_1_, *h*_2_,…, *h*_*n*_) = ⨁_*j*=1_^*n*^(*μ*(*A*_*σ*(*j*)_) − *μ*(*A*_*σ*(*j*−1)_)*h*_*σ*(*j*)_ is called the hesitant triangular fuzzy Choquet ordered averaging (HTFCOA) operator [34].

## 3. Hesitant Triangular Fuzzy Geometric Bonferroni Mean

Based on Definition 5, we can easily verify the following distributive properties.

**Theorem 10 .**

Let *h*_1_, *h*_2_ be two HTFEs; then

1. *λ*(*h*_1_ ⊕ *h*_2_) = *λh*_1_ ⊕ *λh*_2_;
2. (*h*_1_ ⊗ *h*_2_)^*λ*^ = *h*_1_^*λ*^ ⊗ *h*_2_^*λ*^.

We can extend the GBM operator to hesitant triangular fuzzy set.

**Definition 11 .**

Let *h*_*i*_  (*i* = 1,2,…, *n*) be a collection of HTFEs. For any *p*, *q* ≥ 0, if

(5) HTFGBMp,q(h1,h2,…,hn)  =1p+q(⨂i,j=1,i≠jn(phi⊕qhj)1/n(n−1)),

then HTFGBM^*p*,*q*^ is called the hesitant triangular fuzzy geometric Bonferroni mean (HTFGBM).

Especially, if hesitant triangular fuzzy set reduces to hesitant fuzzy set, then the HTFGBM reduces to the I-revised geometric Bonferroni mean developed by Sun and Liu [23].

Based on the operational laws of HTFEs, we can derive the following easy-to-prove theorem whose proof is omitted.

**Theorem 12 .**

Let *p*, *q* ≥ 0 and let *h*_*j*_  (*j* = 1,2,…, *n*) be a collection of HTFEs; then the aggregated value by using the HTFGBM is also a HTFE and

(6) HTFGBMp,q(h1,h2,…,hn)=1p+q(⨂i,j=1,i≠jn(phi⊕qhj)1/n(n−1))=⋃γi∈hi,γj∈hj{(1−(1−∏i,j=1,i≠jn(1−(1−γiL)p(1−γjL)q)1/n(n−1))1/(p+q),1−(1−∏i,j=1,i≠jn(1−(1−γiM)p(1−γjM)q)1/n(n−1))1/(p+q),1−(1−∏i,j=1,i≠jn(1−(1−γiR)p(1−γjR)q)1/n(n−1))1/(p+q))}.

In order to capture the connections between two hesitant fuzzy elements (HFEs), Zhu et al. [22, 35] constructed the hesitant Bonferroni element (HBE) and hesitant fuzzy geometric Bonferroni element (HFGBE), which can be used as calculation units. Inspired by this idea, we can rewrite HTFGBM in another way.

**Theorem 13 .**

Let *p*, *q* ≥ 0 and let *h*_*i*_  (*i* = 1,2,…, *n*) be a collection of HTFEs; then the HTFGBM can be rewritten as

(7) HTFGBMp,q(h1,h2,…,hn)  =1p+q⨂i,j=1,i<jn((phi⊕qhj)⊗(phj⊕qhi))1/n(n−1).

From Definition 11, its proof is straightforward.

Based on the operational laws of HTFEs, we can derive the following theorem.

**Theorem 14 .**

Let *p*, *q* ≥ 0 and let *h*_*i*_  (*i* = 1,2,…, *n*) be a collection of HTFEs; then the aggregated value by using the HTFGBM is a HTFE and

(8) HTFGBMp,q(h1,h2,…,hn)  =⋃ɛij∈τij,i<j{1−(1−∏i,j=1;i<jnɛij1/n(n−1))1/(p+q)},

where *τ*_*ij*,*i*<*j*_ = (*ph*_*i*_ ⊕ *qh*_*j*_)⊗(*ph*_*j*_ ⊕ *qh*_*i*_), *i*, *j* = 1,2,…, *n*.

**Proof**

By the operational laws of HTFEs and Theorem 10, we get *τ*_*ij*,*i*<*j*_ = (*ph*_*i*_ ⊕ *qh*_*j*_)⊗(*ph*_*j*_ ⊕ *qh*_*i*_) is also a HTFE and

(9) $$\begin{array}{c} {\text{HTFGBM}}^{p,q}\left(h_{1},h_{2},\ldots ,h_{n}\right)=\frac{1}{p+q}\overset{n}{\underset{i,j=1,i<j}{⨂}}\tau_{ij}^{1/n(n-1)}. \end{array}$$

By Theorem 10, we further obtain

(10) ⨂i,j=1,i<jnτij1/n(n−1)  =(⨂i,j=1,i<jnτij)1/n(n−1)  =⋃ɛij∈τij,i<j{(∏i,j=1;i<jnɛij)1/n(n−1)}  =⋃ɛij∈τij,i<j{∏i,j=1;i<jnɛij1/n(n−1)}.

Thus

(11) HTFGBMp,q(h1,h2,…,hn)  =1p+q⨂i,j=1,i<jnτij1/n(n−1)  =⋃ɛij∈τij,i<j{1−(1−∏i,j=1;i<jnɛij1/n(n−1))1/(p+q)},

where

(12) ɛij=(phi⊕qhj)⊗(phj⊕qhi)=⋃γi∈hi,γj∈hj{(1−(1−γiL)p(1−γjL)q,1−(1−γiM)p(1−γjM)q,1−(1−γiR)p(1−γjR)q)} ⊗⋃γi∈hi,γj∈hj{(1−(1−γiL)q(1−γjL)p,1−(1−γiM)q(1−γjM)p,1−(1−γiR)q(1−γjR)p)}.

Here, we call *τ*_*ij*_ a hesitant triangular fuzzy geometric Bonferroni element (HTFGBE). The HTFGBE can take much more information into account and can fully represent the connections between two HTFEs. As the basic calculation unit of the HTFGBM, HTFGBE has some desirable properties as follows. As their proofs are straightforward, we omit them here.

**Proposition 15 .**

Let *h*_*α*_*i*__ and *h*_*β*_*i*__ be two collections of HTFEs, *τ*_*α*_*ij*,*i*<*j*__ = (*ph*_*α*_*i*__ ⊕ *qh*_*α*_*j*__)⊗(*ph*_*α*_*j*__ ⊕ *qh*_*α*_*i*__) and *τ*_*β*_*ij*,*i*<*j*__ = (*ph*_*β*_*i*__ ⊕ *qh*_*β*_*j*__)⊗(*ph*_*β*_*j*__ ⊕ *qh*_*β*_*i*__). If, for any *γ*_*α*_*i*__ ∈ *h*_*α*_*i*__ and *γ*_*β*_*j*__ ∈ *h*_*β*_*j*__  (*i*, *j* = 1,2,…, *n*; *i* ≠ *j*), we have *γ*_*α*_*i*__ ≤ *γ*_*β*_*i*__ and *γ*_*α*_*j*__ ≤ *γ*_*β*_*j*__, then *τ*_*α*_*ij*,*i*<*j*__ ≤ *τ*_*β*_*ij*,*i*<*j*__.

**Proposition 16 .**

Let *h*_*i*_  (*i* = 1,2,…, *n*) be a collection of HTFEs, *τ*_*ij*,*i*<*j*_ = (*ph*_*i*_ ⊕ *qh*_*j*_)⊗(*ph*_*j*_ ⊕ *qh*_*i*_) and *h*_*i*_^−^ = ∪_*γ*_*i*_∈*h*_*i*__(min⁡⁡{*γ*_*i*_^*L*^}, min⁡⁡{*γ*_*i*_^*M*^}, min⁡⁡{*γ*_*i*_^*R*^}), *h*_*i*_^+^ = ∪_*γ*_*i*_∈*h*_*i*__(max⁡⁡{*γ*_*i*_^*L*^}, max⁡⁡{*γ*_*i*_^*M*^}, max⁡⁡{*γ*_*i*_^*R*^}), *i*, *j* ∈ {1,2,…, *n*}; then

(13) ⋃γ−∈hi−((1−(1−γ−L)p+q)2,(1−(1−γ−M)p+q)2,(1−(1−γ−R)p+q)2)  ≤τij,i<j  ≤⋃γ+∈hi+((1−(1−γ+L)p+q)2,(1−(1−γ+M)p+q)2,       (1−(1−γ+R)p+q)2).

**Proposition 17 .**

Exchanging *p* and *q*, we have *τ*_*ij*,*i*<*j*_ = (*ph*_*i*_ ⊕ *qh*_*j*_)⊗(*ph*_*j*_ ⊕ *qh*_*i*_) = (*qh*_*i*_ ⊕ *ph*_*j*_)⊗(*qh*_*j*_ ⊕ *ph*_*i*_).

This indicates that the parameters *p* and *q* are symmetric in *HTFGBE*.

**Proposition 18 .**

If one takes *h* = *h*_*i*_ = *h*_*j*_ = {(0,0, 0)} and *h* = *h*_*i*_ = *h*_*j*_ = {(1,1, 1)}, respectively, the corresponding results are *τ*_*ij*,*i*<*j*_ = {(0,0, 0)} or *τ*_*ij*,*i*<*j*_ = {(1,1, 1)}.

Based on the studies above, we can investigate some basic properties of HTFGBM as below.

**Theorem 19 (Monotonicity).**

Let *h*_*α*_*i*__ and *h*_*β*_*i*__  (*i* = 1,2,…, *n*) be two collections of HTFEs; if, for any *γ*_*α*_*i*__ ∈ *h*_*α*_*i*__ and *γ*_*β*_*j*__ ∈ *h*_*β*_*j*__  (*i*, *j* = 1,2,…, *n*; *i* ≠ *j*), one has *γ*_*α*_*i*__ ≤ *γ*_*β*_*i*__ and *γ*_*α*_*j*__ ≤ *γ*_*β*_*j*__, then

(14) HTFGBMp,q(hα1,hα2,…,hαn)  ≤HTFGBMp,q(hβ1,hβ2,…,hβn).

**Proof**

By Proposition 15, we get *ɛ*_*α*_*ij*__ ≤ *ɛ*_*β*_*ij*__, *i*, *j* ∈ {1,2,…, *n*}, *i* ≠ *j*.

Then

(15) 1−(1−∏i,j=1;i≠jnɛαij1/n(n−1))1/(p+q)  ≤1−(1−∏i,j=1;i≠jnɛβij1/n(n−1))1/(p+q).

By Definition 6, we acquire

(16) HTFGBMp,q(hα1,hα2,…,hαn)=1p+q⨂i,j=1,i<jnταij1/n(n−1)=⋃ɛαij∈τij,i<j{1−(1−∏i,j=1;i<jnɛαij1/n(n−1))1/(p+q)}≤⋃ɛβij∈τij,i<j{1−(1−∏i,j=1;i<jnɛβij1/n(n−1))1/(p+q)}=1p+q⨂i,j=1,i<jnτβij1/n(n−1)=HTFGBMp,q(hβ1,hβ2,…,hβn).

**Theorem 20 (boundness).**

Let *h*_*i*_  (*i* = 1,2,…, *n*) be a collection of HTFEs, *h*_*i*_^−^ = ∪_*γ*_*i*_∈*h*_*i*__(min⁡⁡{*γ*_*i*_^*L*^}, min⁡⁡{*γ*_*i*_^*M*^}, min⁡⁡{*γ*_*i*_^*R*^}), and *h*_*i*_^+^ = ∪_*γ*_*i*_∈*h*_*i*__(max⁡⁡{*γ*_*i*_^*L*^}, max⁡⁡{*γ*_*i*_^*M*^}, max⁡⁡{*γ*_*i*_^*R*^}), *i*, *j* ∈ {1,2,…, *n*}; then

(17) ⋃γ−∈hi−(1−(1−(1−(1−γ−L)p+q)2)1/(p+q),1−(1−(1−(1−γ−M)p+q)2)1/(p+q),1−(1−(1−(1−γ−R)p+q)2)1/(p+q)) ≤HTFGBMp,q(h1,h2,…,hn) ≤⋃γ+∈hi+(1−(1−(1−(1−γ+L)p+q)2)1/(p+q),1−(1−(1−(1−γ+M)p+q)2)1/(p+q),1−(1−(1−(1−γ+R)p+q)2)1/p+q).

**Proof**

By Proposition 16, we have

(18) ⋃γ−∈hi−((1−(1−γ−L)p+q)2,(1−(1−γ−M)p+q)2,   (1−(1−γ−R)p+q)2)  ≤ɛij,i<j  ≤⋃γ+∈hi+((1−(1−γ+L)p+q)2,       (1−(1−γ+M)p+q)2,       (1−(1−γ+R)p+q)2).

So

(19) (1−(1−(1−(1−γ−L)p+q)2)1/(p+q),1−(1−(1−(1−γ−M)p+q)2)1/(p+q),1−(1−(1−(1−γ−R)p+q)2)1/(p+q))  ≤1−(1−∏i,j=1;i≠jɛij,i<j1/n(n−1))1/(p+q)  ≤(1−(1−(1−(1−γ+L)p+q)2)1/(p+q),     1−(1−(1−(1−γ+M)p+q)2)1/(p+q),     1−(1−(1−(1−γ+R)p+q)2)1/(p+q)).

By Definition 6, we complete the proof.

**Theorem 21 (commutativity).**

Let *h*_*i*_  (*i* = 1,2,…, *n*) be a collection of HTFEs and let $\left(\tilde{h_{1}},\tilde{h_{2}},\ldots ,\tilde{h_{n}}\right)$ be any permutation of (*h*_1_, *h*_2_,…, *h*_*n*_); then

(20) HTFGBMp,q(h1,h2,…,hn)  =1(p+q)⨂i,j=1,i<jnτij1/n(n−1)  =1(p+q)⨂i,j=1,i<jnτij~1/n(n−1)  =HTFGBMp,q(h1~,h2~,…,hn~),

where *τ*_*ij*,*i*<*j*_ = (*ph*_*i*_ ⊕ *qh*_*j*_)⊗(*ph*_*j*_ ⊕ *qh*_*i*_) and ${\tilde{\tau }}_{ij,i<j}=(p\tilde{h_{i}}⊕q\tilde{h_{j}})⊗(p\tilde{h_{j}}⊕q\tilde{h_{i}})$, *i*, *j* ∈ {1,2,…, *n*}.

**Theorem 22 .**

Let *h*_*i*_  (*i* = 1,2,…, *n*) be a collection of HTFEs; then

(21) $$\begin{array}{c} {HTFGBM}^{p,q}\left(h_{1},h_{2},\ldots ,h_{n}\right)={HTFGBM}^{q,p}\left(h_{1},h_{2},\ldots ,h_{n}\right). \end{array}$$

**Theorem 23 .**

Let *h*_*i*_  (*i* = 1,2,…, *n*) be a collection of HTFEs; if *h*_1_ = *h*_2_ = ⋯ = *h*_*n*_ = *h* = {(0,0, 0)}, one has *HTFGBM* ^*p*,*q*^(*h*_1_, *h*_2_,…, *h*_*n*_) = {(0,0, 0)}. If *h*_1_ = *h*_2_ = ⋯ = *h*_*n*_ = *h* = {(1,1, 1)}, then *HTFGBM* ^*p*,*q*^(*h*_1_, *h*_2_,…, *h*_*n*_) = {(1,1, 1)}.

## 4. Families of Hesitant Triangular Fuzzy Aggregation Operators Based on Bonferroni Means

In practical society, the decision-makers may have different needs. In order to meet the different needs, we develop various hesitant triangular fuzzy aggregation operators based on Bonferroni means in this section. As their properties are similar to HTFGBM, we omit them for the sake of simplicity.

Based on Definition 9, we can develop hesitant triangular fuzzy Bonferroni mean as below.

**Definition 24 .**

Let *h*_*i*_  (*i* = 1,2,…, *n*) be a collection of HTFEs. For any *p*, *q* ≥ 0, one calls

(22) HTFBMp,q(h1,h2,…,hn)  =(1n(n−1)⨁i,j=1,i≠jn(hip⊗hjq))1/(p+q)

the hesitant triangular fuzzy Bonferroni mean (HTFBM).

**Remark 25 .**

Especially, if hesitant triangular fuzzy set reduces to triangular fuzzy set, then HTFBM reduces to the triangular fuzzy Bonferroni mean developed by Zhu et al. [24]. Furthermore, let *p* = 1, *q* = 0; then HTFBM reduces to HTFBM^1,0^(*h*_1_, *h*_2_,…, *h*_*n*_) = (1/*n*)(⊕_*i*=1_^*n*^ *h*_*i*_). Besides, if hesitant triangular fuzzy set reduces to hesitant fuzzy set, then HTFBM reduces to the hesitant fuzzy Bonferroni mean proposed by Zhu and Xu [22].

In some practical applications, we have to weight the hesitant triangular fuzzy arguments. Then, by giving weights to each attribute, we can develop the weighted operators as below.

**Definition 26 .**

Let *h*_*i*_  (*i* = 1,2,…, *n*) be a collection of HTFEs, *p*, *q* ≥ 0, and *w* = (*w*_1_, *w*_2_,…, *w*_*n*_)^*T*^ the weight of *h*_*i*_, where *w*_*i*_ denotes the importance degree of *h*_*i*_, satisfying *w*_*i*_ > 0 and ∑_*i*=1_^*n*^ *w*_*i*_ = 1. Then

(23) HTFWGBMp,q(h1,h2,…,hn)  =1p+q(⨂i,j=1,i≠jn(phiwi⊕qhjwj)1/n(n−1)),HTFWBMp,q(h1,h2,…,hn)  =(1n(n−1)⨁i,j=1,i≠jn(wihip⊗wjhjq))1/(p+q)

are called the hesitant triangular fuzzy weighted geometric Bonferroni mean (HTFWGBM) and the hesitant triangular fuzzy weighted Bonferroni mean (HTFWBM), respectively.

**Remark 27 .**

Suppose there is only one triangular fuzzy value in each *h*_*i*_  (*i* = 1,2,…, *n*) and let *p* = 1, *q* = 0; then HTFWGBM^1,0^(*h*_1_, *h*_2_,…, *h*_*n*_) = ⨂_*i*=1_^*n*^(*h*_*i*_^*w*_*i*_^)^1/*n*^ = (HTFWG(*h*_1_, *h*_2_,…, *h*_*n*_))^1/*n*^ and HTFWBM^1,0^(*h*_1_, *h*_2_,…, *h*_*n*_) = (1/*n*)⨁_*i*=1_^*n*^(*w*_*i*_ *h*_*i*_) = (1/*n*)HTFWA(*h*_1_, *h*_2_,…, *h*_*n*_).

Sometimes, we may need to weight the ordered positions of the hesitant triangular fuzzy arguments instead of weighting the arguments themselves. In this case, we can develop the ordered weighted operators as follows.

**Definition 28 .**

Let *h*_*i*_  (*i* = 1,2,…, *n*) be a collection of HTFEs, *p*, *q* ≥ 0, and *w* = (*w*_1_, *w*_2_,…, *w*_*n*_)^*T*^ the associated weight vector such that *w*_*i*_ > 0 and ∑_*i*=1_^*n*^ *w*_*i*_ = 1. (*σ*(1), *σ*(2),…, *σ*(*n*)) is a permutation of (1,2,…, *n*), such that *h*_*σ*(*j*−1)_ ≥ *h*_*σ*(*j*)_ for all *j* = 2,3,…, *n*. Then

(24) HTFOWGBMp,q(h1,h2,…,hn)  =1p+q(⨂i,j=1,i≠jn(phσ(i)wi⊕qhσ(j)wj)1/n(n−1)),HTFOWBMp,q(h1,h2,…,hn)  =(1n(n−1)⨁i,j=1,i≠jn(wihσ(i)p⊗wjhσ(j)q))1/(p+q)

are called the hesitant triangular fuzzy ordered weighted geometric Bonferroni mean (HTFOWGBM) and the hesitant triangular fuzzy ordered weighted Bonferroni mean (HTFOWBM), respectively.

**Remark 29 .**

Suppose there is only one triangular fuzzy value in each *h*_*i*_  (*i* = 1,2,…, *n*) and let *p* = 1, *q* = 0; then HTFOWGBM^1,0^(*h*_1_, *h*_2_,…, *h*_*n*_) = ⨂_*i*=1_^*n*^(*h*_*σ*(*i*)_^*w*_*i*_^)^1/*n*^ = (HTFOWG(*h*_1_, *h*_2_,…, *h*_*n*_))^1/*n*^ and HTFOWBM^1,0^(*h*_1_, *h*_2_,…, *h*_*n*_) = (1/*n*)⨁_*i*=1_^*n*^(*w*_*i*_ *h*_*σ*(*i*)_) = (1/*n*)HTFOWA(*h*_1_, *h*_2_,…, *h*_*n*_). If *h*_1_ ≥ *h*_2_ ≥ ⋯≥*h*_*n*_, then HTFOWGBM and HTFOWBM reduce to HTFWGBM and HTFWBM, respectively.

Inspired by Xu [36], when we want to not only weight the hesitant triangular fuzzy arguments but also weight the ordered positions of the hesitant triangular fuzzy arguments, we can propose the following hybrid average operators.

**Definition 30 .**

Let *h*_*i*_  (*i* = 1,2,…, *n*) be a collection of HTFEs, *p*, *q* ≥ 0, and *w* = (*w*_1_, *w*_2_,…, *w*_*n*_)^*T*^ the associated weight vector such that *w*_*i*_ > 0 and ∑_*i*=1_^*n*^ *w*_*i*_ = 1. Let ${\tilde{h}}_{\sigma (j)}$ be the *j*th largest element of the hesitant triangular fuzzy arguments (${\tilde{h}}_{j}={\left(h_{j}\right)}^{nw_{j}},\;j=1,2,\ldots ,n$). Then, one calls

(25) HTFHGBMp,q(h1,h2,…,hn)  =1p+q(⨂i,j=1,i≠jn(ph~σ(i)wi⊕qh~σ(j)wj)1/n(n−1))

the hesitant triangular fuzzy hybrid geometric Bonferroni mean (HTFHGBM).

**Definition 31 .**

Let *h*_*i*_  (*i* = 1,2,…, *n*) be a collection of HTFEs, *p*, *q* ≥ 0, and *w* = (*w*_1_, *w*_2_,…, *w*_*n*_)^*T*^ the associated weight vector such that *w*_*i*_ > 0 and ∑_*i*=1_^*n*^ *w*_*i*_ = 1. Let ${\tilde{h}}_{\sigma (j)}$ be the *j*th largest element of the hesitant triangular fuzzy arguments (${\tilde{h}}_{j}=(nw_{j})h_{j},j=1,2,\ldots ,n$). Then, one calls

(26) HTFHBMp,q(h1,h2,…,hn)  =(1n(n−1)⨁i,j=1,i≠jn(wih~σ(i)p⊗wjh~σ(j)q))1/(p+q)

the hesitant triangular fuzzy hybrid Bonferroni mean (HTFHBM).

**Remark 32 .**

If there is only one triangular fuzzy value in each *h*_*i*_  (*i* = 1,2,…, *n*) and letting *p* = 1, *q* = 0, then $\text{HTFHGB}{\text{M}}^{1,0}(h_{1},h_{2},\ldots ,h_{n})={⨂}_{i=1}^{n}{\left({\tilde{h}}_{\sigma (i)}^{w_{i}}\right)}^{1/n}={\left(\text{HTFHG}(h_{1},h_{2},\ldots ,h_{n})\right)}^{1/n}$, $\text{HTFHB}{\text{M}}^{1,0}(h_{1},h_{2},\ldots ,h_{n})=\left(1/n\right)\left({⨁}_{i=1}^{n}(w_{i}{\tilde{h}}_{\sigma (i)})\right)=\left(1/n\right)\text{HTFHA}(h_{1},h_{2},\ldots ,h_{n})$.

However, the above aggregation operators are based on the assumption that the attributes are independent. In real decision-making problems, these is usually interaction among attributes. As we all know, the Choquet integral [37] can depict the correlations of attributes. Combining the BM and the Choquet integral, Zhu et al. [35] developed a hesitant fuzzy Choquet geometric Bonferroni mean. Motivated by their idea, we develop the hesitant triangular fuzzy Choquet ordered Bonferroni mean as follows.

**Definition 33 .**

Let *h*_*i*_  (*i* = 1,2,…, *n*) be a collection of HTFEs on *X*, *μ* a fuzzy measure on *X*, and *p*, *q* ≥ 0. Then, one calls

(27) HTFCOBMμp,q(h1,h2,…,hn)  =(1n(n−1)⨁i,j=1,i≠jn((μ(Aσ(i))−μ(Aσ(i−1)))hσ(i)p    ⊗(μ(Aσ(j))−μ(Aσ(j−1)))hσ(j)q))1/(p+q)

the hesitant triangular fuzzy Choquet ordered Bonferroni mean (HTFCOBM), where (*σ*(1), *σ*(2),…, *σ*(*n*)) is a permutation of (1,2,…, *n*), such that *h*_*σ*(*j*−1)_ ≥ *h*_*σ*(*j*)_ for all *j* = 2,3,…, *n*, *A*_*σ*(*k*)_ = {*x*_*σ*(*j*)_∣*j* ≤ *k*}, for *k* ≥ 1, and *A*_*σ*(0)_ = *ϕ*.

**Remark 34 .**

If *μ*({*x*_*σ*(*j*)_}) = *μ*({*A*_*σ*(*j*)_}) − *μ*({*A*_*σ*(*j*−1)_}), *j* = 1,2,…, *n*, then HTFCOBM reduces to HTFWBM. Let *w*_*j*_ = *μ*({*A*_*σ*(*j*)_}) − *μ*({*A*_*σ*(*j*−1)_}), *j* = 1,2,…, *n*, then HTFCOBM reduces to HTFOWBM. In addition, suppose there is only one triangular fuzzy value in each *h*_*i*_  (*i* = 1,2,…, *n*) and let *p* = 1, *q* = 0, then HTFCOBM_*μ*_^1,0^(*h*_1_, *h*_2_,…, *h*_*n*_) = (1/*n*)(⨁_*i*=1_^*n*^(*μ*(*A*_*σ*(*i*)_) − *μ*(*A*_*σ*(*i*−1)_)*h*_*σ*(*i*)_) = (1/*n*)HTFCOA_*μ*_(*h*_1_, *h*_2_,…, *h*_*n*_). This is the so-called hesitant triangular fuzzy Choquet ordered averaging operator proposed by Zhong and Xu [34].

## 5. An Approach to Multiple Attribute Decision Making with Hesitant Triangular Fuzzy Information

In this section, we shall utilize the proposed operators to multiple attribute decision-makings under hesitant triangular fuzzy environment. As their procedures are similar, we only consider the HTFCOBM operator here.

The following assumptions or notations are used to represent the MADM problems for evaluation of theses with hesitant triangular fuzzy information. Let *A* = {*A*_1_, *A*_2_,…, *A*_*m*_} be a set of *m* alternatives and *G* = {*G*_1_, *G*_2_,…, *G*_*n*_} a set of *n* attributes. If the decision-makers provide values for the alternative *A*_*i*_ under the attribute *G*_*j*_ with anonymity, these values can be considered as a hesitant triangular fuzzy element *h*_*ij*_. In the case where two decision-makers provide the same value, the value emerges only once in *h*_*ij*_. Suppose that the decision matrix *H* = (*h*_*ij*_)_*m*×*n*_ is the hesitant triangular fuzzy decision matrix, where *h*_*ij*_  (*i* = 1,2,…, *m*, *j* = 1,2,…, *n*) are in the form of HTFEs.

In the following, we apply the HTFCOBM operator to the MADM problems for evaluation of theses with hesitant triangular fuzzy information.

*Step 1.* Confirm the fuzzy measures *μ* of attributes of *G* and attributes sets of *G*. 

*Step 2.* We utilize the decision information given in matrix *H* and the HTFCOBM operator

(28) h~k=HTFCOBMμp,q(hk1,hk2,…,hkn)=(1n(n−1)⨁i,j=1,i≠jn((μ(Aσ(i))−μ(Aσ(i−1)))hσ(ki)p  ⊗(μ(Aσ(j))−μ(Aσ(j−1)))hσ(kj)q))1/(p+q)

to derive the overall preference values ${\tilde{h}}_{k}\;\;(k=1,2,\ldots ,m)$ of the alternative *A*_*k*_. 

*Step 3.* Calculate the scores $S({\tilde{h}}_{k})\;\;(k=1,2,\ldots ,m)$ of the overall hesitant triangular fuzzy values ${\tilde{h}}_{k}$ by Definition 6. 

*Step 4.* Compare each $S({\tilde{h}}_{i})$ with all the $S({\tilde{h}}_{j})\;\;(i,j=1,2,\ldots ,m)$ by Definition 4. For convenience, we let $p_{ij}=p(S({\tilde{h}}_{i})\geq S({\tilde{h}}_{j}))$; then we develop a complementary matrix as *P* = (*p*_*ij*_)_*m*×*m*_, where *p*_*ij*_ ≥ 0, *p*_*ij*_ + *p*_*ji*_ = 1, *p*_*ii*_ = 0.5, *i*, *j* = 1,2,…, *m*. Summing all the elements in each line of matrix *P*, we have *p*_*i*_ = ∑_*j*=1_^*m*^ *p*_*ij*_, *i* = 1,2,…, *m*.

*Step 5.* Rank all the alternatives *A*_*i*_  (*i* = 1,2,…, *m*) in accordance with the values of *p*_*i*_ and select the best one(s). 

*Step 6.* End.

**Remark 35 .**

The advantages of our method lie in four aspects.

First, with the aid of fuzzy measure *μ*, the HTFCOBM operator can deal with the situation where the attributes are correlative. The weight vectors can be obtained by the source decision information in our method. Traditional additive aggregation operators, such as HTFWA and HTFWG operators, are all based on the assumption that the attributes are independent and each attribute is given a fixed weight representing its importance during the decision process. As a result, they cannot get reasonable results when the attributes are correlative.

Second, as we all know, the desirable characteristic of the BM is its ability to capture the interrelation among the input arguments. As a result, the HTFCOBM operator can deal with the situation where the input arguments are correlative.

Third, the HTFCOBM operator can accommodate situations in which the input arguments are hesitant triangular fuzzy information. As hesitant triangular fuzzy set is a comprehensive set containing FS and HFS as special cases, our method can be widely used.

Fourth, the HTFCOBM operator has additional parameters *p*, *q* which control the power. If the parameters take different values, the HTFCOBM operator can be viewed as extensions of some exiting operators under certain conditions. The decision-makers can choose different parameters according to their preferences and interests, which makes decision-making more flexible.

## 6. Numerical Example

In this section, we will present a numerical example (adapted from [38]) to show evaluation of theses with hesitant triangular fuzzy information in order to illustrate the proposed method.

Suppose there are five theses *A*_*i*_  (*i* = 1,2, 3,4, 5) and we want to select the best one. Four attributes are selected by experts to evaluate the theses: (1) *G*_1_ is the language of a thesis; (2) *G*_2_ is the innovation; (3) *G*_3_ is the rigor; (4) *G*_4_ is the structure of the thesis. Perhaps the author who has accurate language also pays great attention to rigorous reasoning. That is to say, there are interactions between these attributes. In order to avoid influencing each other, the experts are required to evaluate the five theses *A*_*i*_  (*i* = 1,2, 3,4, 5) under the above four attributes in anonymity and the decision matrix *H* = (*h*_*ij*_)_5×4_ is presented in Table 1, where *h*_*ij*_  (*i* = 1,2, 3,4, 5, *j* = 1,2, 3,4) are in the form of HTFEs. In the review process, if the thesis has beautiful language, an expert may give better score to the structure of the thesis due to the previous good impression. In other words, there are interrelationships between input arguments. Thus, the HTFCOBM operator is a good choice here. The fuzzy measure of attribute *G*_*j*_  (*j* = 1,2,…, 4) and attribute sets of *G* are as follows: *μ*(*G*_1_) = 0.30, *μ*(*G*_2_) = 0.35, *μ*(*G*_3_) = 0.30, *μ*(*G*_4_) = 0.22, *μ*(*G*_1_, *G*_2_) = 0.70, *μ*(*G*_1_, *G*_3_) = 0.60, *μ*(*G*_1_, *G*_4_) = 0.55, *μ*(*G*_2_, *G*_3_) = 0.50, *μ*(*G*_2_, *G*_4_) = 0.45, *μ*(*G*_3_, *G*_4_) = 0.40, *μ*(*G*_1_, *G*_2_, *G*_3_) = 0.82, *μ*(*G*_1_, *G*_2_, *G*_4_) = 0.87, *μ*(*G*_1_, *G*_3_, *G*_4_) = 0.75, *μ*(*G*_2_, *G*_3_, *G*_4_) = 0.60, and *μ*(*G*_1_, *G*_2_, *G*_3_, *G*_4_) = 1.00.

### 6.1. The Decision-Making Steps

Next, we apply the developed approach to evaluate these theses with hesitant triangular fuzzy information.

*Step 1.* We use the decision information given in matrix *H* and the HTFCOBM operator (here, we take *p* = *q* = 1) to obtain the overall preference values ${\tilde{h}}_{i}$ of the thesis *A*_*i*_  (*i* = 1,2, 3,4, 5). Due to the large amount of data, we omitted these results here. When assigning different values to the parameters *p* and *q*, we can obtain different results. Please see Table 2. 

*Step 2.* Calculate the scores $S({\tilde{h}}_{i})\;(i=1,2,3,4,5)$ of the overall hesitant triangular fuzzy preference values ${\tilde{h}}_{i}$ by Definition 6: $S({\tilde{h}}_{1})=(0.096,0.134,0.211),S({\tilde{h}}_{2})=(0.132,0.180,0.261),S({\tilde{h}}_{3})=$ $\left(0.083,0.142,0.209),S({\tilde{h}}_{4})=(0.106,0.180,0.262),S({\tilde{h}}_{5})=(0.121,0.181,0.287\right)$. 

*Step 3.* Comparing each $S({\tilde{h}}_{i})$ with all the $S({\tilde{h}}_{j})\;\;(i,j=1,2,\ldots ,5)$ by Definition 4, then we develop a complementary matrix as *P* = (*p*_*ij*_)_5×5_. Summing all the elements in each line of the matrix *P*, we have *p*_1_ = 1.478, *p*_2_ = 3.277, *p*_3_ = 1.558, *p*_4_ = 2.924, *p*_5_ = 3.263. 

*Step 4.* Rank all the alternatives *A*_*i*_  (*i* = 1,2,…, *m*) in accordance with the values of *p*_*i*_: *A*_2_≻*A*_5_≻*A*_4_≻*A*_3_≻*A*_1_. Note that ≻ means “preferred to.” Thus, the best thesis is *A*_2_.

### 6.2. Discussion

**Figure 1:**
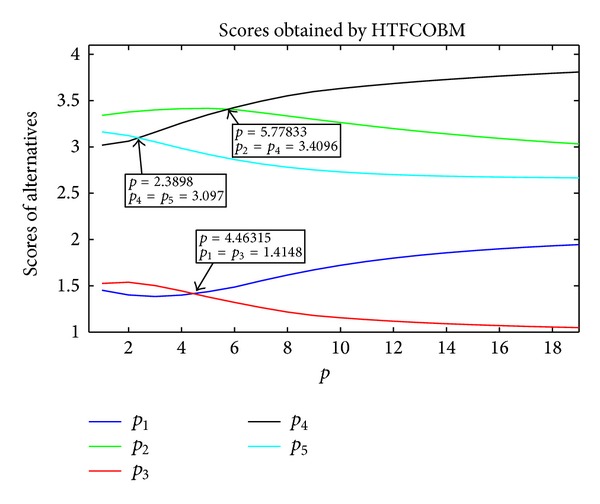
Scores for theses obtained by HTFCOBM operator.

From Table 2, we find that the values obtained by the HTFCOBM operator change as the parameters *p*, *q* vary. Moreover, the rankings are different when we choose different values of *p*, *q*. As the two parameters are symmetrical, we can fix one of them and change the other. Here, we set *q* = 2, for example. The trends are shown in Figure 1 as the parameter *p* ranges from 0 to 18. From Figure 1, it can be clearly seen that

1. when *p* ∈ (0,2.3898], the ranking of the five theses is *A*_2_≻*A*_5_≻*A*_4_≻*A*_3_≻*A*_1_;
2. when *p* ∈ (2.3898,4.46315], the ranking of the five theses is *A*_2_≻*A*_4_≻*A*_5_≻*A*_3_≻*A*_1_;
3. when *p* ∈ (4.46315,5.77833], the ranking of the five theses is *A*_2_≻*A*_4_≻*A*_5_≻*A*_1_≻*A*_3_;
4. when *p* ∈ (5.77833,18], the ranking of the five theses is *A*_4_≻*A*_2_≻*A*_5_≻*A*_1_≻*A*_3_.

Apparently, different parameters can be chosen according to decision-makers' interests, which makes the decision more flexible.

### 6.3. Comparative Analysis

In order to show the merit of the proposed method, we utilized some existing methods proposed by Wei et al. [15] and Zhong and Xu [34] to solve this illustrate example. For simplicity, we omit the calculation process and only list the results in Tables 3 and 4.

From Tables 2, 3, and 4, we can compare these methods as follows.

(1) During the calculation, we can find that the weight vectors can be obtained by the source decision information in our method. As a result, different decision data will acquire different weight vectors automatically. However, for other operators such as HTFWA and HTFWG, the weight vectors must be given by experts in advance. Thus, the proposed method is more reasonable and objective.

(2) Comparing Tables 3 and 4, we can find the relations such as

(29) HTFWGBM1,0(h31,h32,h33,,h34)  =(HTFWG(h31,h32,h33,,h34))1/4.

In this case, we can view HTFWG as a special case of HTFWGBM. This has been mentioned in Section 4.

(3) From Table 4, we can find the relations such as

(30) HTFOWBM1,1(h31,h32,h33,,h34)  =HTFWBM1,1(h31,h32,h33,,h34).

Thus, the HTFOWBM can reduce to HTFWBM under certain conditions, which has also been pointed out in Section 4.

(4) We find that the rankings in Table 3 are different from Table 4. The reason may be that there are interdependent phenomena among attributes or input arguments in this numerical example. For example,

(31) μ(G1)+μ(G2)+μ(G3)+μ(G4)  =0.30+0.35+0.30+0.22>1  =μ(G1,G2,G3,G4)

also tells us that the attributes are correlative. The HTFCOBM operator can perform aggregation of attributes when they are correlative and it allows argument values to support each other in the aggregation process. However, the existing operators, such as HTFWA and HTFWG, always suppose that the attributes are independent and each attribute is given a fixed weight subjectively. So the HTFCOBM operator is a better choice here.

(5) When we change the parameters *p*, *q*, we get different rankings in Table 2. This indicates that the HTFCOBM operator can meet the needs of different types of decision-makers.

## 7. Conclusion

In this paper, we have investigated the multiple attribute decision-making (MADM) problems based on the HTFCOBM operator with hesitant triangular fuzzy information. Firstly, some basic concepts related to hesitant triangular fuzzy set have been reviewed. Then, motivated by the ideal of BM and Choquet integral, some new hesitant triangular fuzzy aggregation operators such as HTFCOBM have been developed. The prominent advantage of HTFCOBM is that it can consider the correlations between the attributes and among the input arguments, which makes it more feasible and practical. At the same time, we have discussed their basic properties. As different parameters can be chosen in these new operators, the decision becomes more flexible. Furthermore, we have discussed the families of new operators. Under certain conditions, they can be seen as extensions of the existing operators. Next, we have applied the HTFCOBM operator to multiple attribute decision-making problems in which attribute values take the form of hesitant triangular fuzzy information. Finally, an illustrative example for evaluation of theses has been given to demonstrate the proposed method. There are some other generalizations of Bonferroni mean such as the generalized hesitant fuzzy Bonferroni mean [21] and normalized geometric Bonferroni operators [23], which can also be used to construct new operators for hesitant triangular fuzzy set. The researches on these new operators may be interesting and meaningful.

## Figures and Tables

**Table 1:** Hesitant triangular fuzzy decision matrix *H*.

	*G*_1_	*G*_2_	*G*_3_	*G*_4_
*A*_1_	{(0.2, 0.3, 0.6), (0.3, 0.4, 0.6)}	{(0.2,0.3,0.5)}	{(0.6,0.7,0.8)}	{(0.3,0.4,0.5)}
*A*_2_	{(0.2, 0.3, 0.5), (0.4, 0.5, 0.6), (0.5, 0.6, 0.7)}	{(0.6,0.7,0.9)}	{(0.2,0.4,0.5)}	{(0.6,0.7,0.8)}
*A*_3_	{(0.4,0.6,0.8)}	{(0.4,0.5,0.6)}	{(0.2,0.4,0.6)}	{(0.1,0.3,0.4)}
*A*_4_	{(0.2, 0.4, 0.5), (0.3, 0.6, 0.8)}	{(0.1,0.2,0.3)}	{(0.4, 0.6, 0.7), (0.5, 0.7, 0.9)}	{(0.7,0.8,0.9)}
*A*_5_	{(0.3,0.5,0.7)}	{(0.4,0.5,0.7)}	{(0.2, 0.5, 0.6), (0.5, 0.6, 0.7)}	{(0.6,0.7,0.9)}

**Table 2:** Scores for alternatives obtained by HTFCOBM operator.

(*p*, *q*)	$s({\tilde{h}}_{1})$	$s({\tilde{h}}_{2})$	$s({\tilde{h}}_{3})$	$s({\tilde{h}}_{4})$	$s({\tilde{h}}_{5})$	Ranking
(1,0)	(0.110, 0.151, 0.230)	(0.148, 0.198, 0.304)	(0.096, 0.156, 0.231)	(0.118, 0.191, 0.280)	(0.124, 0.186, 0.300)	*A*_2_≻*A*_5_≻*A*_4_≻*A*_3_≻*A*_1_
(1,1)	(0.096, 0.134, 0.211)	(0.132, 0.180, 0.261)	(0.083, 0.142, 0.209)	(0.106, 0.180, 0.262)	(0.121, 0.181, 0.287)	*A*_2_≻*A*_5_≻*A*_4_≻*A*_3_≻*A*_1_
(1,3)	(0.194, 0.250, 0.351)	(0.250, 0.314, 0.421)	(0.169, 0.256, 0.352)	(0.221, 0.321, 0.425)	(0.224, 0.306, 0.441)	*A*_2_≻*A*_4_≻*A*_5_≻*A*_1_≻*A*_3_
(2,6)	(0.292, 0.361, 0.472)	(0.360, 0.434, 0.555)	(0.250, 0.358, 0.475)	(0.339, 0.452, 0.566)	(0.323, 0.414, 0.564)	*A*_4_≻*A*_2_≻*A*_5_≻*A*_1_≻*A*_3_

**Table 3:** Scores for theses obtained by the exsting operators. (Let *w* = (0.3,0.4,0.12,0.18)^*T*^).

Operators	$s({\tilde{h}}_{1})$	$s({\tilde{h}}_{2})$	$s({\tilde{h}}_{3})$	$s({\tilde{h}}_{4})$	$s({\tilde{h}}_{5})$	Ranking
HTFCOA	(0.373, 0.479, 0.649)	(0.472, 0.585, 0.765)	(0.332, 0.492, 0.651)	(0.394, 0.572, 0.727)	(0.411, 0.561, 0.759)	*A*_2_≻*A*_5_≻*A*_4_≻*A*_1_≻*A*_3_
HTFWA	(0.295, 0.399, 0.581)	(0.503, 0.615, 0.793)	(0.332, 0.492, 0.651)	(0.342, 0.512, 0.668)	(0.412, 0.550, 0.750)	*A*_2_≻*A*_5_≻*A*_4_≻*A*_3_≻*A*_1_
HTFWG	(0.261, 0.366, 0.559)	(0.447, 0.574, 0.726)	(0.287, 0.469, 0.608)	(0.223, 0.387, 0.515)	(0.384, 0.537, 0.726)	*A*_2_≻*A*_5_≻*A*_3_≻*A*_1_≻*A*_4_
HTFOWA	(0.389, 0.494, 0.630)	(0.522, 0.637, 0.792)	(0.332, 0.492, 0.651)	(0.481, 0.643, 0.792)	(0.450, 0.577, 0.780)	*A*_2_≻*A*_4_≻*A*_5_≻*A*_1_≻*A*_3_
HTFOWG	(0.335, 0.442, 0.588)	(0.461, 0.600, 0.735)	(0.287, 0.469, 0.608)	(0.364, 0.541, 0.674)	(0.418, 0.559, 0.748)	*A*_2_≻*A*_5_≻*A*_4_≻*A*_3_≻*A*_1_
HTFHA	(0.304, 0.412, 0.592)	(0.547, 0.654, 0.829)	(0.404, 0.572, 0.736)	(0.371, 0.555, 0.707)	(0.451, 0.572, 0.778)	*A*_2_≻*A*_5_≻*A*_3_≻*A*_4_≻*A*_1_
HTFHG	(0.338, 0.442, 0.604)	(0.468, 0.589, 0.765)	(0.322, 0.506, 0.666)	(0.334, 0.498, 0.619)	(0.465, 0.611, 0.771)	*A*_5_≻*A*_2_≻*A*_3_≻*A*_4_≻*A*_1_

**Table 4:** Scores for theses obtained by new operators based on BM or GBM.

New operators	$s({\tilde{h}}_{1})$	$s({\tilde{h}}_{2})$	$s({\tilde{h}}_{3})$	$s({\tilde{h}}_{4})$	$s({\tilde{h}}_{5})$	Ranking
HTFGBM^1,1^	(0.331, 0.436, 0.603)	(0.440, 0.569, 0.711)	(0.269, 0.450, 0.605)	(0.365, 0.545, 0.682)	(0.409, 0.563, 0.742)	*A*_2_≻*A*_5_≻*A*_4_≻*A*_1_≻*A*_3_
HTFBM^1,1^	(0.320, 0.426, 0.598)	(0.427, 0.563, 0.705)	(0.256, 0.444, 0.597)	(0.339, 0.525, 0.674)	(0.404, 0.561, 0.739)	*A*_2_≻*A*_5_≻*A*_4_≻*A*_1_≻*A*_3_
HTFWGBM^1,1^	(0.745, 0.800, 0.875)	(0.824, 0.876, 0.928)	(0.737, 0.832, 0.889)	(0.750, 0.839, 0.890)	(0.801, 0.866, 0.928)	*A*_2_≻*A*_5_≻*A*_4_≻*A*_3_≻*A*_1_
HTFWBM^1,1^	(0.083, 0.118, 0.189)	(0.130, 0.182, 0.260)	(0.072, 0.133, 0.198)	(0.087, 0.153, 0.226)	(0.114, 0.173, 0.274)	*A*_2_≻*A*_5_≻*A*_4_≻*A*_3_≻*A*_1_
HTFOWGBM^1,1^	(0.773, 0.825, 0.887)	(0.828, 0.881, 0.929)	(0.737, 0.832, 0.889)	(0.789, 0.867, 0.916)	(0.811, 0.873, 0.934)	*A*_2_≻*A*_5_≻*A*_4_≻*A*_1_≻*A*_3_
HTFOWBM^1,1^	(0.091, 0.128, 0.195)	(0.130, 0.185, 0.265)	(0.072, 0.133, 0.198)	(0.104, 0.173, 0.250)	(0.118, 0.176, 0.278)	*A*_2_≻*A*_5_≻*A*_4_≻*A*_3_≻*A*_1_
HTHGBM^1,1^	(0.779, 0.828, 0.890)	(0.837, 0.883, 0.937)	(0.759, 0.847, 0.905)	(0.797, 0.871, 0.916)	(0.829, 0.888, 0.940)	*A*_5_≻*A*_2_≻*A*_4_≻*A*_3_≻*A*_1_
HTHBM^1,1^	(0.078, 0.112, 0.178)	(0.128, 0.177, 0.252)	(0.077, 0.136, 0.199)	(0.085, 0.149, 0.218)	(0.112, 0.165, 0.262)	*A*_2_≻*A*_5_≻*A*_4_≻*A*_3_≻*A*_1_
